# Supplementary material for: Arabidopsis root lipid droplets are hubs for membrane homeostasis under heat stress, and triterpenoid synthesis and storage
Source: New Phytol. 2025 Nov 25;249(2):892–916. doi: 10.1111/nph.70726 (PMC12712439; doi:10.1111/nph.70726)
Supplement: Supplementary file 5 — Fig. S1 Lipid droplets are enriched in parts of the elongation zone in Arabidopsis wild‐type seedlings. Fig. S2 Comparison of root LDs from Col‐0 and the tgd1‐1 sdp1‐4 mutant under control conditions. Fig. S3 Comparison of root LDs from Col‐0 and the tgd1‐1 sdp1‐4 mutant under heat stress. Fig. S4 Root LDs are depleted in the early meristematic zone and accumulate under heat stress. Fig. S5 The average number of double bonds decreases in most lipid classes in Arabidopsis seedlings subjected to heat stress. Fig. S6 Composition of glycerolipids in control and heat‐stressed roots from Col‐0 and the mutant tgd1‐1 sdp1‐4. Fig. S7 Abundance of lipids in control and heat‐stressed roots from Col‐0 and the mutant tgd1‐1 sdp1‐4. Fig. S8 Enrichment of different organellar proteomes in the LD‐enriched fraction. Fig. S9 Subcellular localization of GLYCEROL‐3‐PHOSPHATE ACYLTRANSFERASE 4 (GPAT4) in Nicotiana tabacum pollen tubes. Fig. S10 Subcellular localization of Arabidopsis N‐glycan biosynthetic enzymes in Nicotiana benthamiana leaves. Fig. S11 Subcellular localization of selected Arabidopsis candidate root LD proteins in Nicotiana tabacum pollen tubes. Fig. S12 Subcellular localization of Arabidopsis putative dehydrogenases in Nicotiana tabacum pollen tubes. Fig. S13 Subcellular localization of selected candidate Arabidopsis root LDs proteins with unknown function in Nicotiana tabacum pollen tubes and Nicotiana benthamiana leaf cells. Fig. S14 Subcellular localization of candidate Arabidopsis root LD proteins with unknown function in Nicotiana tabacum pollen tubes. Fig. S15 Subcellular localization of various Arabidopsis enzymes acting downstream of thalianol synthase. Fig. S16 Analysis of protein enrichment in the LD fraction of Arabidopsis roots of the mutant tgd1‐1 sdp1‐4 grown in axenic root culture. Fig. S17 Analysis of protein enrichment in the LD fraction of heat‐stressed Arabidopsis roots of the mutant tgd1‐1 sdp1‐4 grown in axenic root culture. Fig. S18 Analysis of p [file NPH-249-892-s002.pdf]

New Phytologist Supporting Information

Article title: Arabidopsis root lipid droplets are hubs for membrane homeostasis under heat stress, and triterpenoid synthesis and storage.

Authors: Patricia Scholz, Janis Dabisch, Ana C. Vilchez, Alyssa C. Clews, Philipp W. Niemeyer, Magdiel S. S. Lim, Siqi Sun, Lea Hembach, Mayuko Naganawa, Fabienne Dreier, Katharina F. Blersch, Lea M. Preuß, Martin Bonin, Elena Lesch, Yuya Iwai, Takashi L. Shimada, Jürgen Eirich, Iris Finkemeier, Katharina Gutbrod, Peter Dörmann, You Wang, Robert T. Mullen, Till Ischebeck

Article acceptance date: 3 October 2025

Supplemental Figures S1-S19

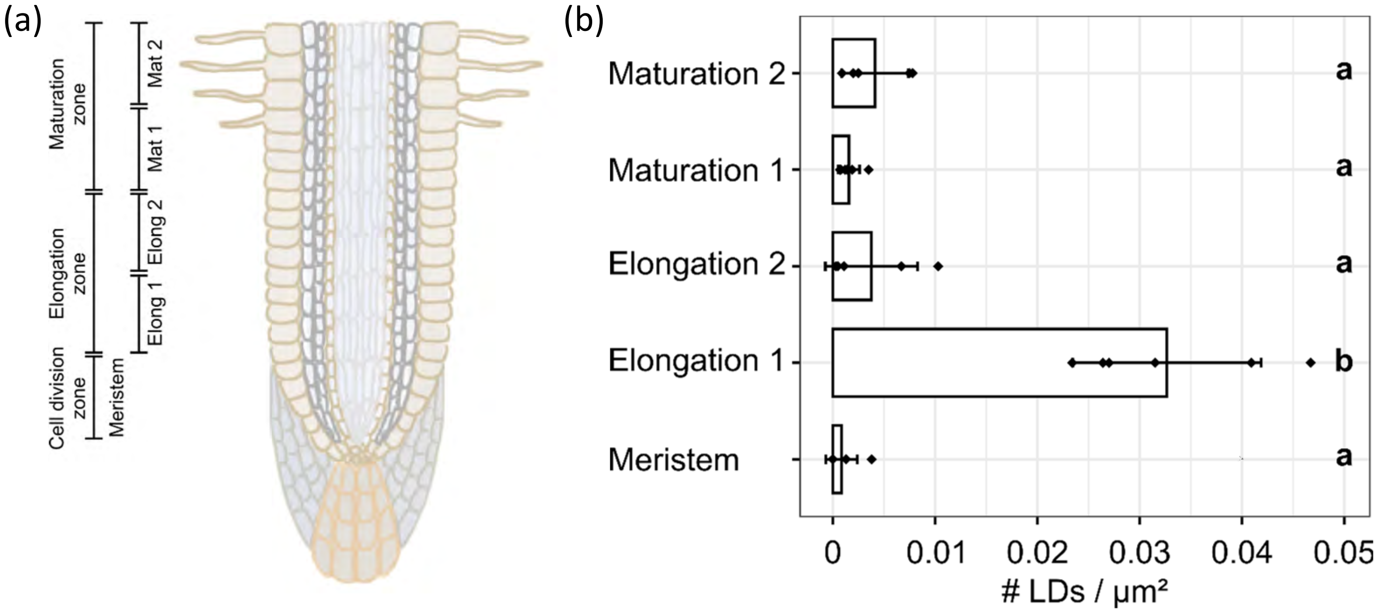

Figure S1: LDs are enriched in different parts of the root elongation zone in Arabidopsis wild-type seedlings. The roots of 7-day-old wild-type seedlings grown vertically on plates (1/2 MS without sucrose) were fixed and stained with BODIPY 493/503. Plants were grown at 23°C prior to analysis. Median planes of different root zones (a) were imaged by CLSM (see Figure 1). For quantitative image analysis, areas up to 100 µm x 100 µm of each root micrograph were selected and the LDs within the selected areas were quantified in number and size using the particle analysis tool of ImageJ. Roots showed a significantly increased number of LDs in the elongation zone 1 compared to other regions of the root (b). Data was analyzed from $n \geq 5$ individual roots. Statistical comparison of the LD number between root zones was carried out using one way ANOVA with post-hoc Tukey test. Different letters indicate significant differences with $p < 0.05$. Plots display mean ± standard deviation. Data is identical to the data of the control presented in Figure 1.

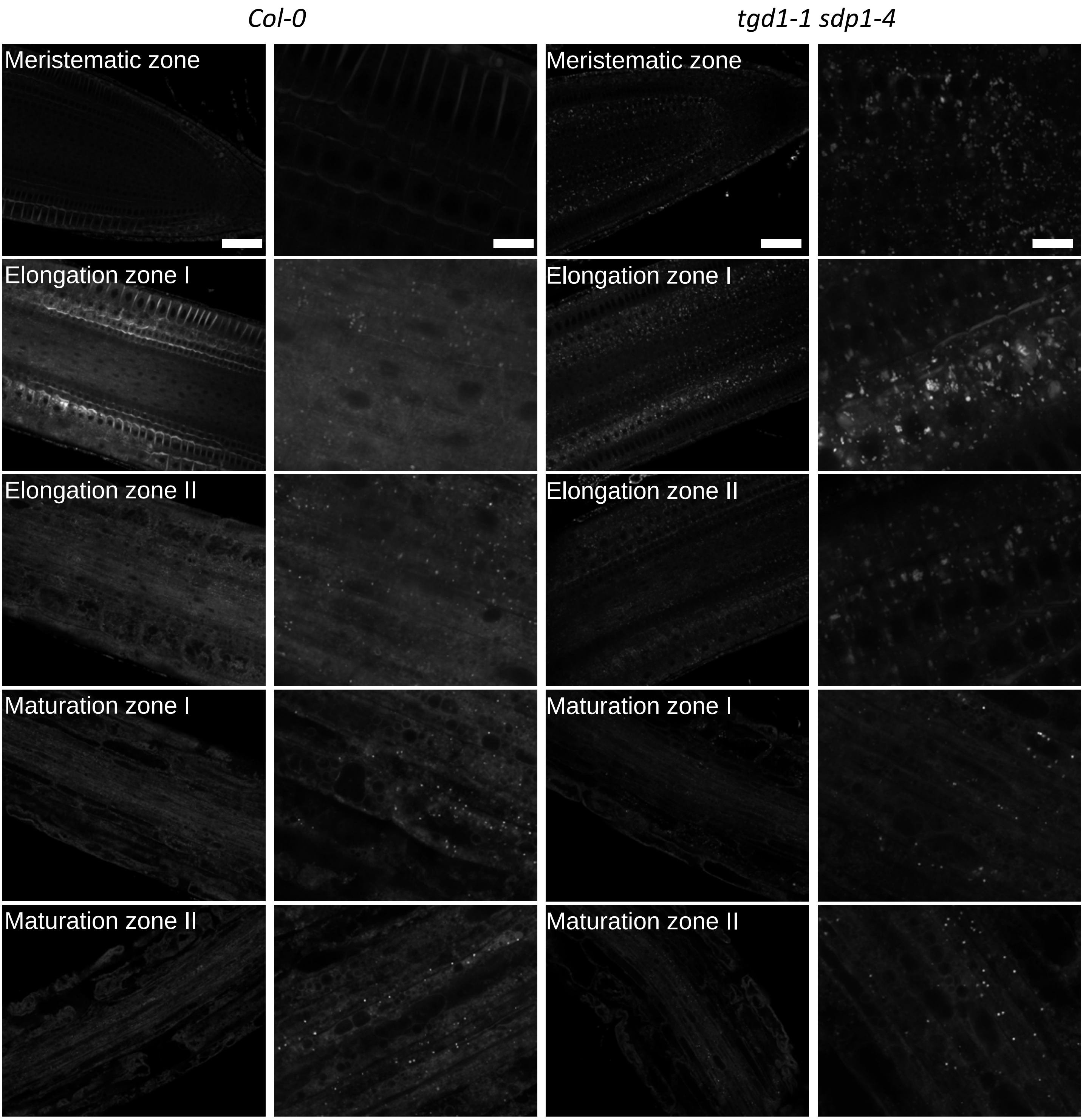

Figure S2: Comparison of Arabidopsis root LDs from Col-0 and the *tgd1-1 sdp1-4* mutant under control conditions. Seedlings were vertically grown on plates containing 1 % (w/v) sucrose for 12 d (Col-0) or 14 days (*tgd1-1 sdp1-4*) to reach the same length and development. The roots were fixed and stained with BODIPY 493/503. Plants were grown at 23°C (this figure) and, in the case of heat stress, moved for 24 h to 37°C prior to fixation and analysis (Figure S3). Median planes of different root zones (see Figure S1) were imaged by CLSM. Images display overviews (1st and 3rd column) and magnifications (2nd and 4th column). Bars, 50 µm and 10 µm, respectively. Images are representative for at least three individual roots per line. For quantitative image analysis, see Figure S4.

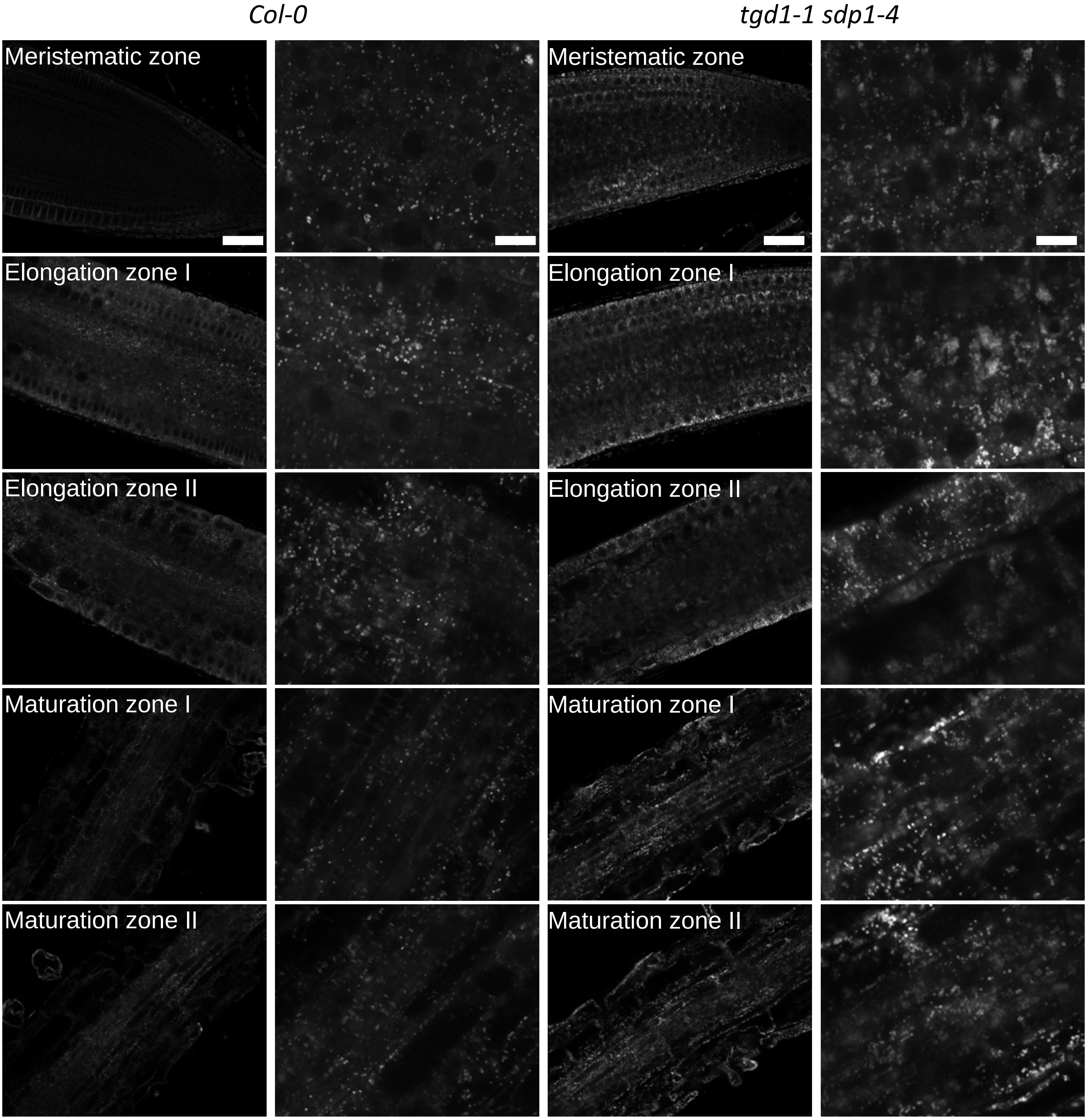

Figure S3: Comparison of Arabidopsis root LDs from Col-0 and the *tgd1-1 sdp1-4* mutant under heat stress. Seedlings were vertically grown on plates containing 1 % (w/v) sucrose for 12 d (Col-0) or 14 days (*tgd1-1 sdp1-4*) to reach the same length and development. The roots were fixed and stained with BODIPY 493/503. Plants were grown at 23°C (Figure S2) and, in the case of heat stress, moved for 24 h to 37°C prior to fixation and analysis (this figure). Median planes of different root zones (see Figure S1) were imaged by CLSM. Images display overviews (1st and 3rd column) and magnifications (2nd and 4th column). Bars, 50 µm and 10 µm, respectively. Images are representative for at least three individual roots per line. For quantitative image analysis, see Figure S4.

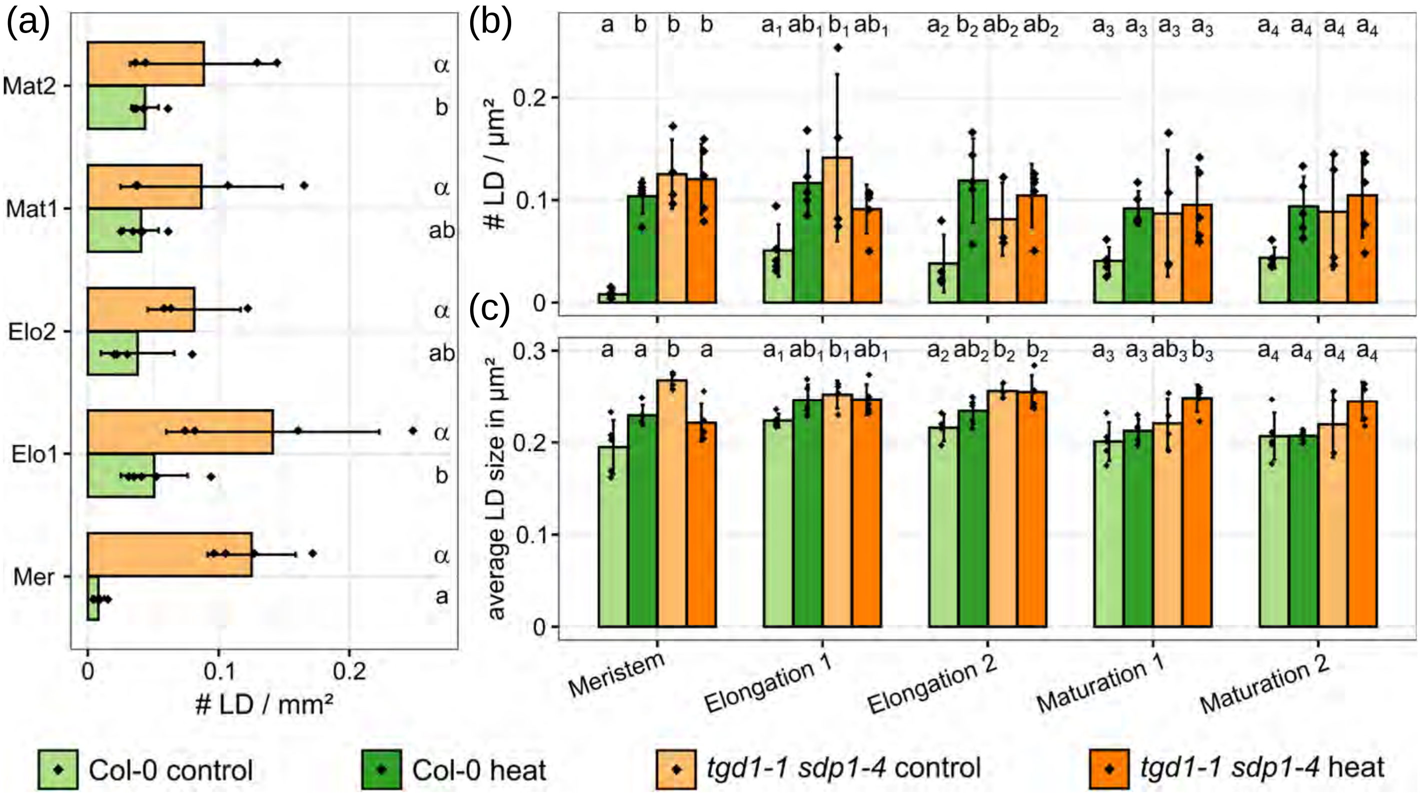

Figure S4: Root LDs are depleted in the early meristematic zone and accumulate under heat stress. Arabidopsis seedlings were vertically grown on plates containing 1 % (w/v) sucrose for 12 d (Col-0) or 14 days (*tgd1-1 sdp1-4*) to reach the same length and development. The roots were fixed and stained with BODIPY 493/503. Plants were grown at 23°C and, in the case of heat stress, moved for 24 h to 37°C prior to fixation and analysis. For quantitative image analysis, LDs were segmented with ilastik 1.4.1post1 and subsequently quantified in Fiji ImageJ. In the indicated root zones, areas across the root section were selected, with a height of ca. 150 µm along the root axis, and LDs quantified in number and size. Root cap cells were omitted from the analysis. Data was analysed from >= 3 individual roots. Two different statistical comparisons were made: For each genotype under control conditions, the number of LDs within the root zone were compared between root zones, using one way ANOVA with post-hoc Tukey test (left plot). In addition, statistical comparisons were performed within root zones, also using one way ANOVA with post-hoc Tukey test (right plots). Plots display mean ± standard deviation.

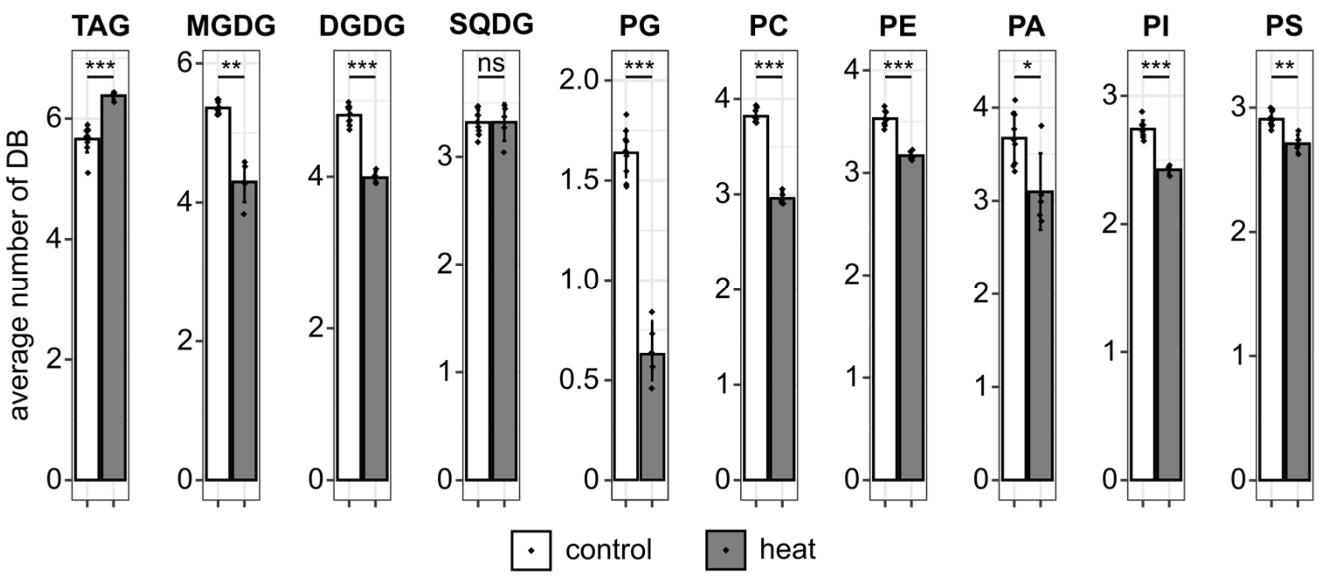

Figure S5: The average number of double bonds decreases in most lipid classes in Arabidopsis seedlings subjected to heat stress. Lipids were extracted from roots of 12-day-old Col-0 seedlings grown vertically on plates. Plants were grown at 23°C, and in the case of heat stress moved for 24 h to 37°C prior to analysis. Lipids were analyzed by ESI-MS/MS. Determination of individual lipid species composition allowed the calculation of the average number of double bonds. Values are from n ≥ 5 biological replicates and are shown as mean ± standard deviation. Statistical differences were calculated by Welch's *t*-test using Benjamini-Hochberg correction for multiple comparisons and are represented as follows: $p > 0.05$ “ns”, $p < 0.05$ “*”, $p < 0.01$ “**”, $p < 0.001$ “***”.

DB, double bonds; DGDG, digalactosyldiacylglycerol; MGDG, monogalactosyldiacylglycerol; PA, phosphatidic acid; PC, phosphatidylcholine, PE, phosphatidylethanolamine; PG, phosphatidylglycerol; PI, phosphatidylinositol; PS, phosphatidylserine; sulfoquinovosyldiacylglycerol; TAG, triacylglycerol.

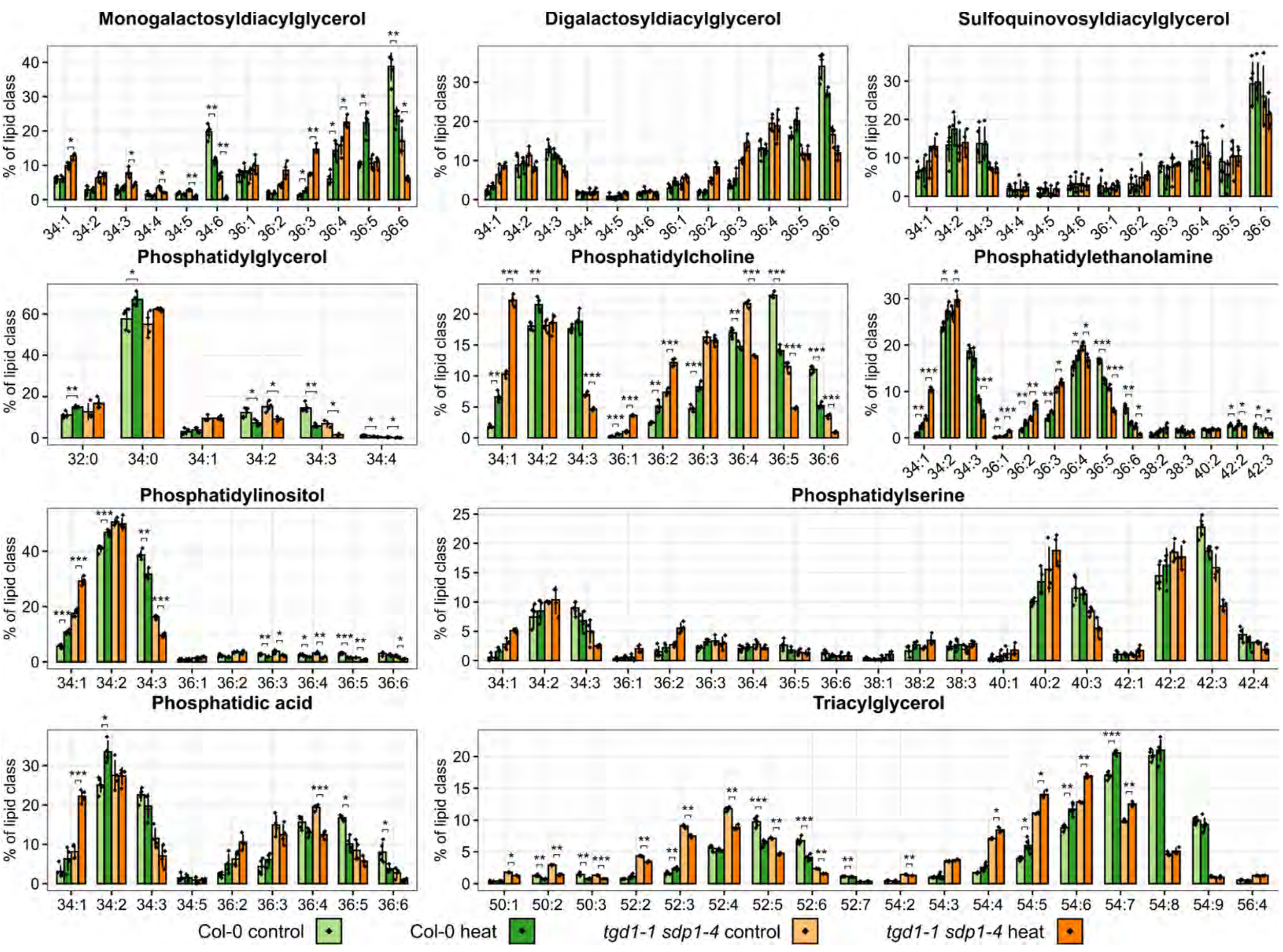

Figure S6: Composition of glycerolipids in control and heat-stressed roots from Col-0 and the mutant *tgd1-1 sdp1-4*. Arabidopsis seedlings were vertically grown on plates containing 1 % (w/v) sucrose for 12 d (Col-0) or 14 days (*tgd1-1 sdp1-4*) to reach the same length and development. Plants were grown at 23°C, and in the case of heat stress moved for 24 h to 37°C prior to analysis. Lipids were extracted from roots and analyzed by ESI-MS/MS. Absolute values for the molar amounts of individual lipid species were determined, and their relative proportion in the respective lipid class was calculated in mol %. Values are from n = 3-5 biological replicates, and are shown as mean ± standard deviation. Statistical differences were calculated by Welch's *t*-test using Benjamini-Hochberg correction for multiple comparisons and are represented as follows: $p > 0.05$ "ns", $p < 0.05$ "*", $p < 0.01$ "**", $p < 0.001$ "***".

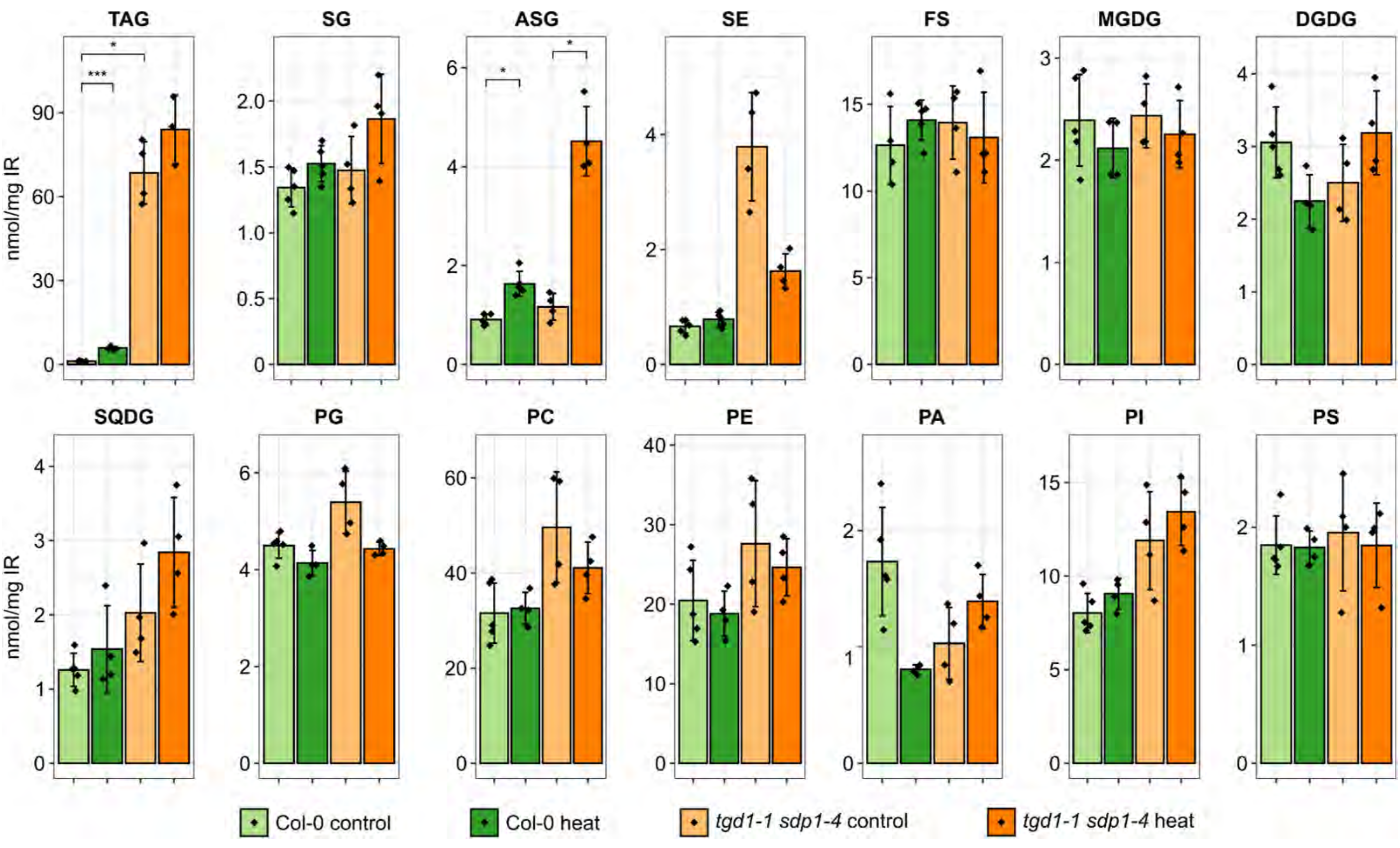

Figure S7: Abundance of lipids in control and heat-stressed roots from Col-0 and the mutant *tgd1-1 sdp1-4*. Arabidopsis seedlings were vertically grown on plates containing 1 % (w/v) sucrose for 12 d (Col-0) or 14 days (*tgd1-1 sdp1-4*) to reach the same length and development. Plants were grown at 23°C, and in the case of heat stress moved for 24 h to 37°C prior to analysis. Lipids were extracted from roots and analyzed by ESI-MS/MS. Total amounts of lipid classes were determined as sums of all individual lipid species of the respective lipids class. Values are from n = 3-5 biological replicates, and are shown as mean ± standard deviation. Statistical differences were calculated by Welch's *t*-test using Benjamini-Hochberg correction for multiple comparisons and are represented as follows: $p > 0.05$ "ns", $p < 0.05$ "*", $p < 0.01$ "**", $p < 0.001$ "***". ASG, acylated steryl glycosides; DGDG, digalactosyldiacylglycerol; FS, free sterols; IR, insoluble residue; MGDG, monogalactosyldiacylglycerol; PA, phosphatidic acid; PC, phosphatidylcholine, PE, phosphatidylethanolamine; PG, phosphatidylglycerol; PI, phosphatidylinositol; PS, phosphatidylserine; SE, sterol esters; SG, steryl glycosides; SQDG, sulfoquinovosyldiacylglycerol; TAG, triacylglycerol.

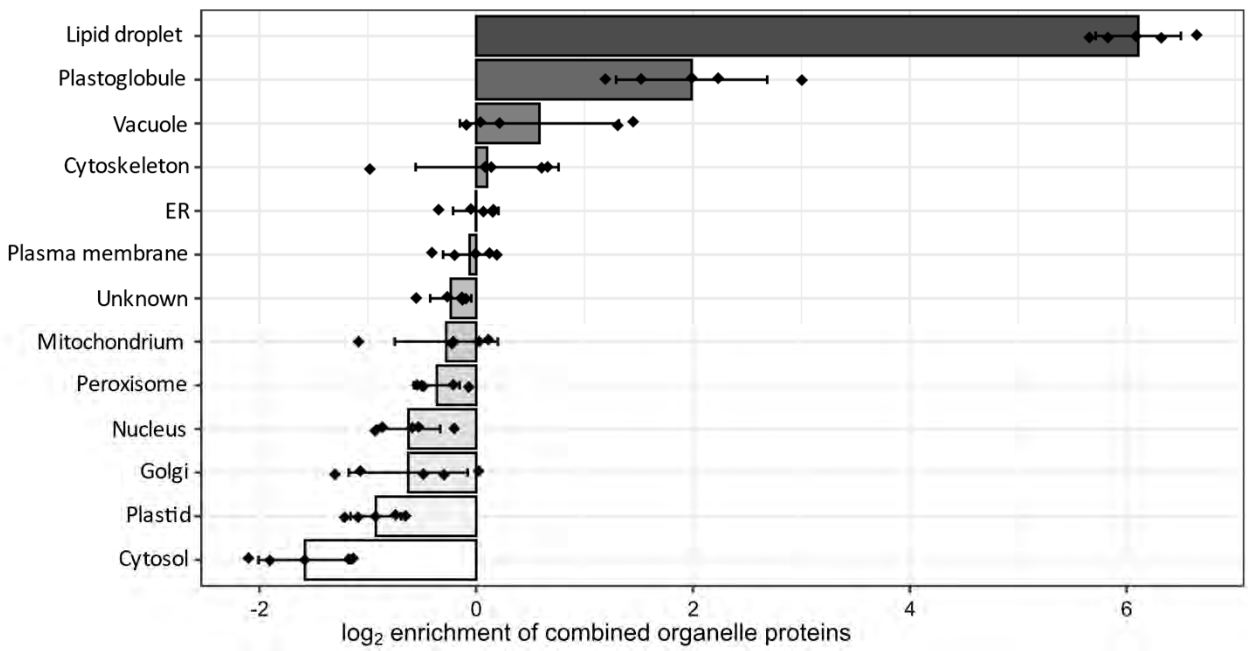

Figure S8: Enrichment of different organellar proteomes in the LD-enriched fraction. Arabidopsis roots of *tgd1-1 sdp1-4* mutant plants were grown in axenic root culture and an LD-enriched fraction isolated. The proteome of this fraction and a total protein fraction was investigated by LC-MS/MS and relative iBAQ values were calculated. To analyze enrichments of different subcellular organelles, proteins were assigned to their subcellular localization according to the online Plant Proteome Database and previous reports of known plant LD proteins. Only proteins identified by at least two peptides and present in all replicates of at least one fraction were used for this analysis. Furthermore, assignment to organelles was only done for proteins with a unique localization, all other proteins were designated as “unknown”. The combined protein abundance of all marker proteins of the different organelles in the LD-fraction was then normalized to the respective combined protein abundances in total extract samples and the resulting enrichment ratio was log_2-transformed. n = 5 biological replicates, values are shown as mean ± standard deviation.

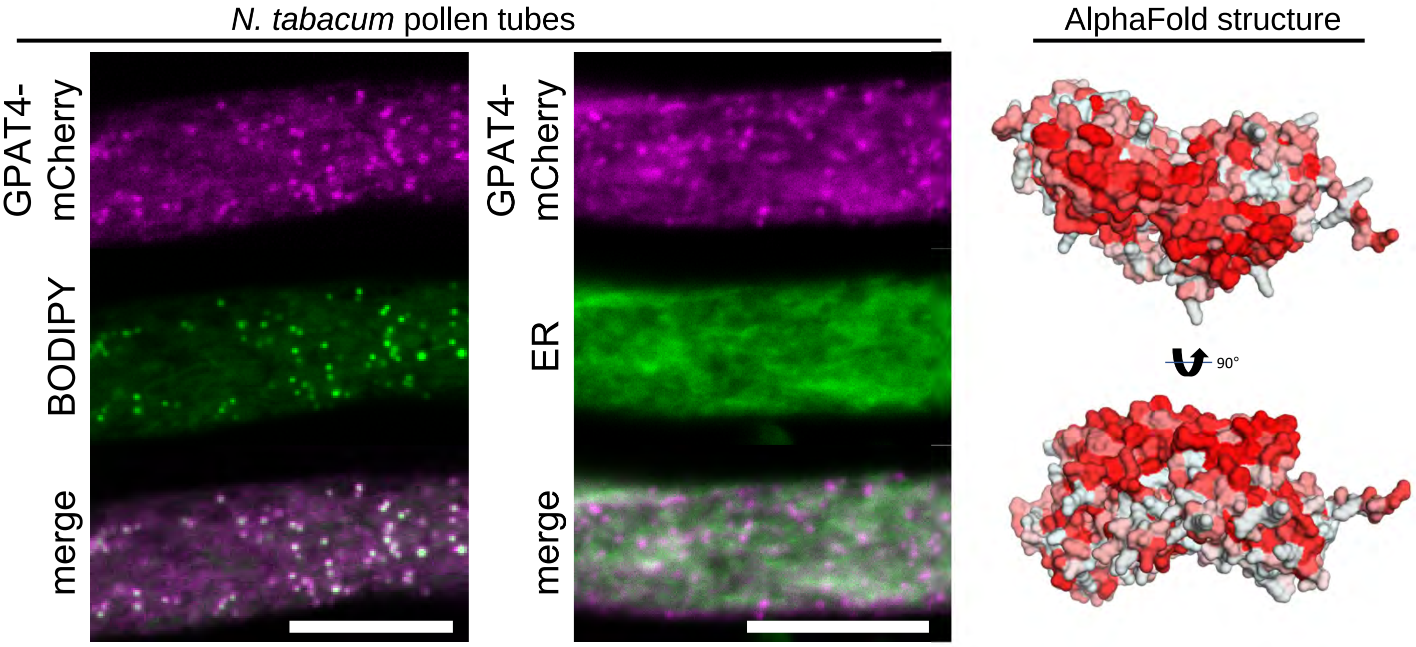

Figure S9: Subcellular localization of Arabidopsis GLYCEROL-3-PHOSPHATE ACYLTRANSFERASE 4 (GPAT4) in *N. tabacum* pollen tubes. mCherry-tagged GPAT4 was expressed in *N. tabacum* pollen tubes. Either LDs were stained with BODIPY 493/503 or the ER marker ERD2-CFP was co-expressed. Images are single planes obtained by CLSM. GPAT4 clearly colocalized with LDs in pollen tubes. Each image is representative for 10 pollen tubes. Bars, 10 µm. As shown on the right, the protein structure of GPAT4, as predicted by AlphaFold2, shows a hydrophobic surface on one side of the protein.

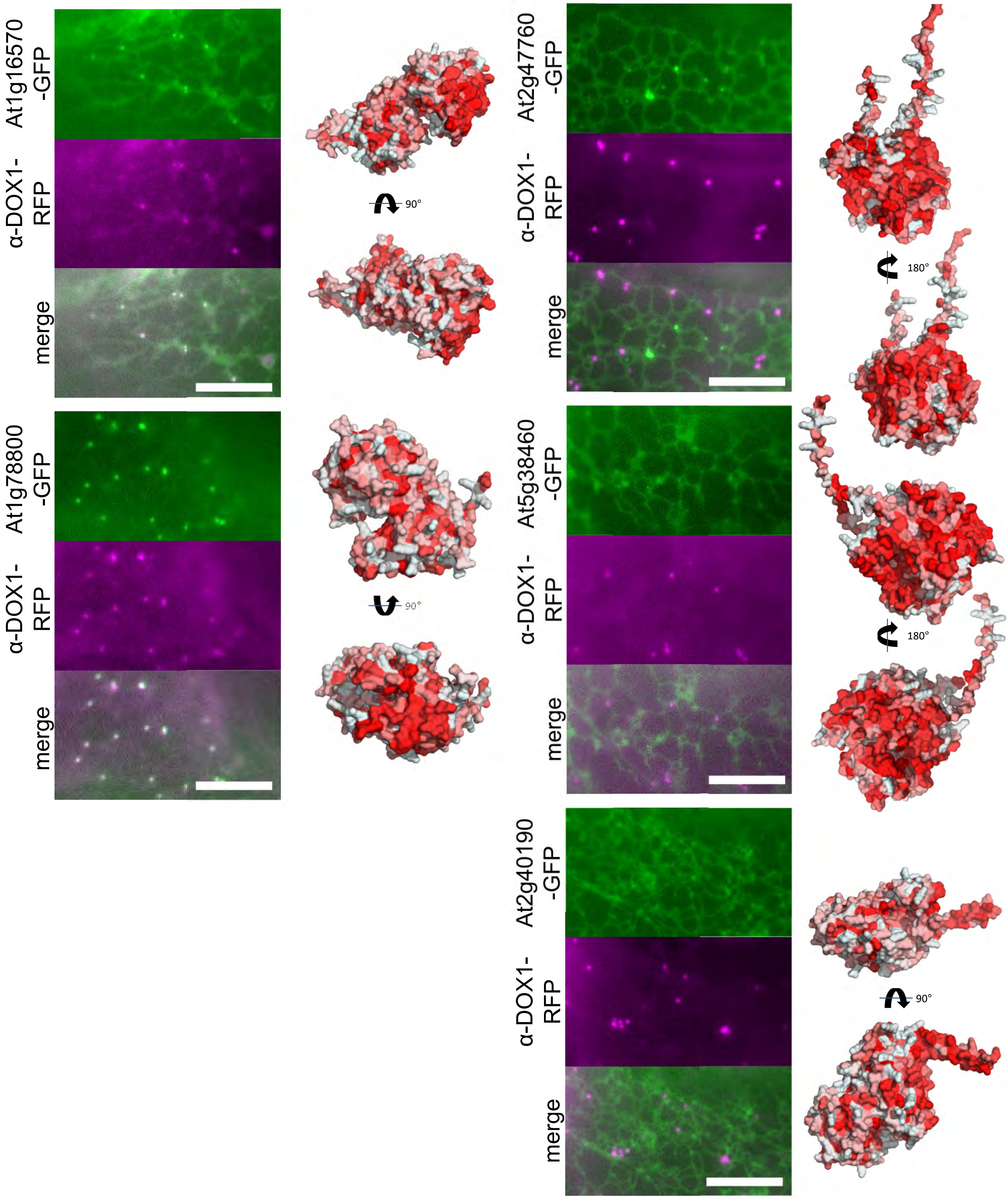

Figure S10: Subcellular localization of Arabidopsis N-glycan biosynthetic enzymes in *N. benthamiana* leaves. Indicated GFP-tagged proteins were expressed in *N. benthamiana* leaves. The formation of LDs was induced by heat stress and α-DOX1-RFP was co-expressed, serving as an LD marker protein. Images were obtained by fluorescence microscope. At1g16570 and At1g78800 colocalize with the LD marker, while the other three members of the protein family display a reticular pattern. Each image is representative for at least 4 leaf areas. Bars, 10 µm. As shown on the right, the protein structures of At1g16570 and At1g78800, as predicted by AlphaFold2, show a hydrophobic surface on one side of the protein.

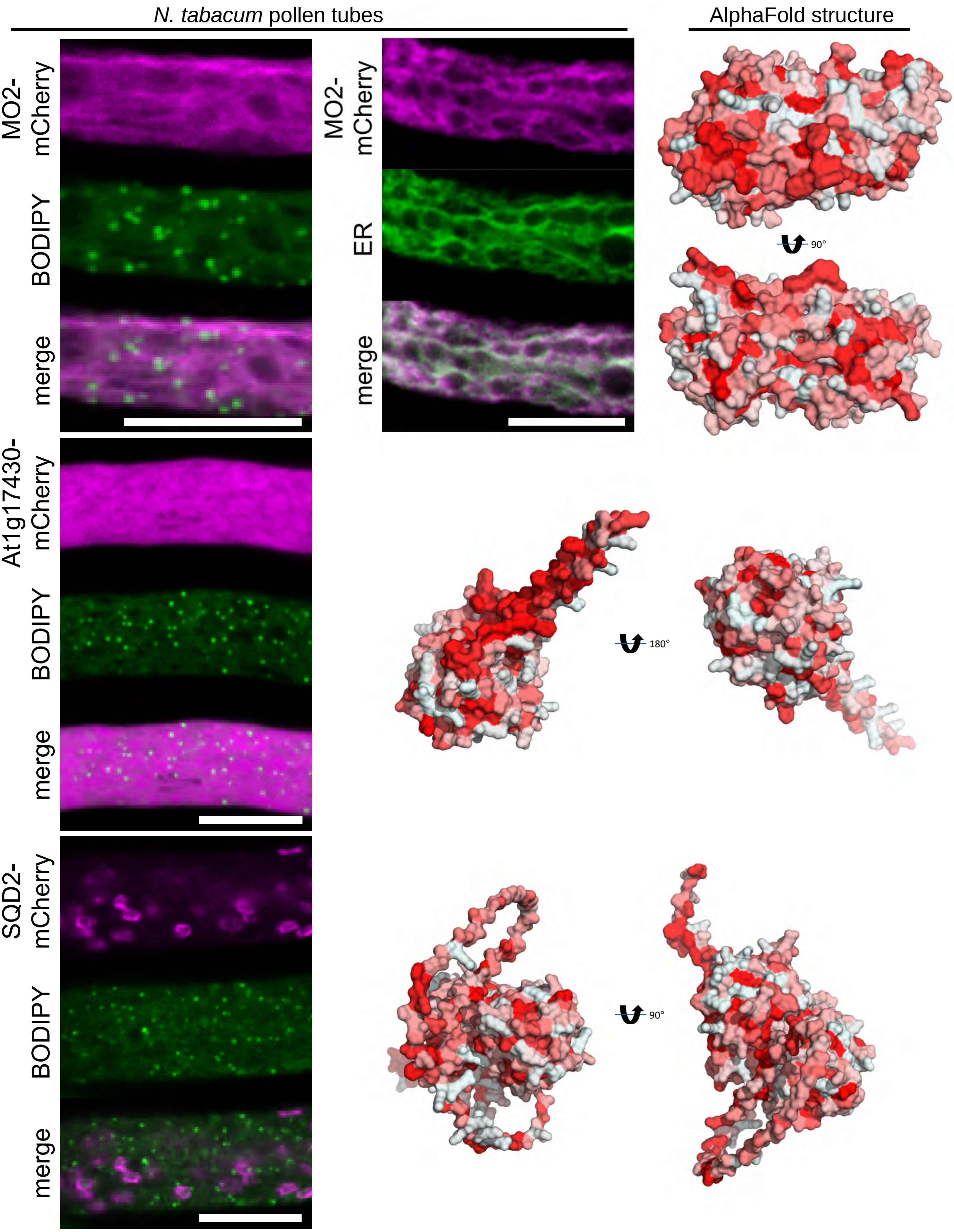

Figure S11: Subcellular localization of selected Arabidopsis candidate root LD proteins in *N. tabacum* pollen tubes. Indicated mCherry-tagged proteins were expressed in *N. tabacum* pollen tubes. Either LDs were stained with BODIPY 493/503 or ERD2-CFP was co-expressed, serving as an ER marker. Images are single planes obtained by CLSM. MONOOXYGENASE 2 (MO2) colocalized with the ER marker while the putative hydrolase At1g17430 displayed cytosolic localization. SULFOQUINOVOSYLDIACYLGLYCEROL 2 (SQD2) localizes to structures with high resemblance to plastids in pollen tubes. Each image is representative for at least 7 pollen tubes. Bars, 10 µm. The top two structures, as predicted by AlphaFold2, show hydrophobic surface regions but not a flat face like involved in several LD-binding proteins. SQD2 does not display any larger hydrophobic regions.

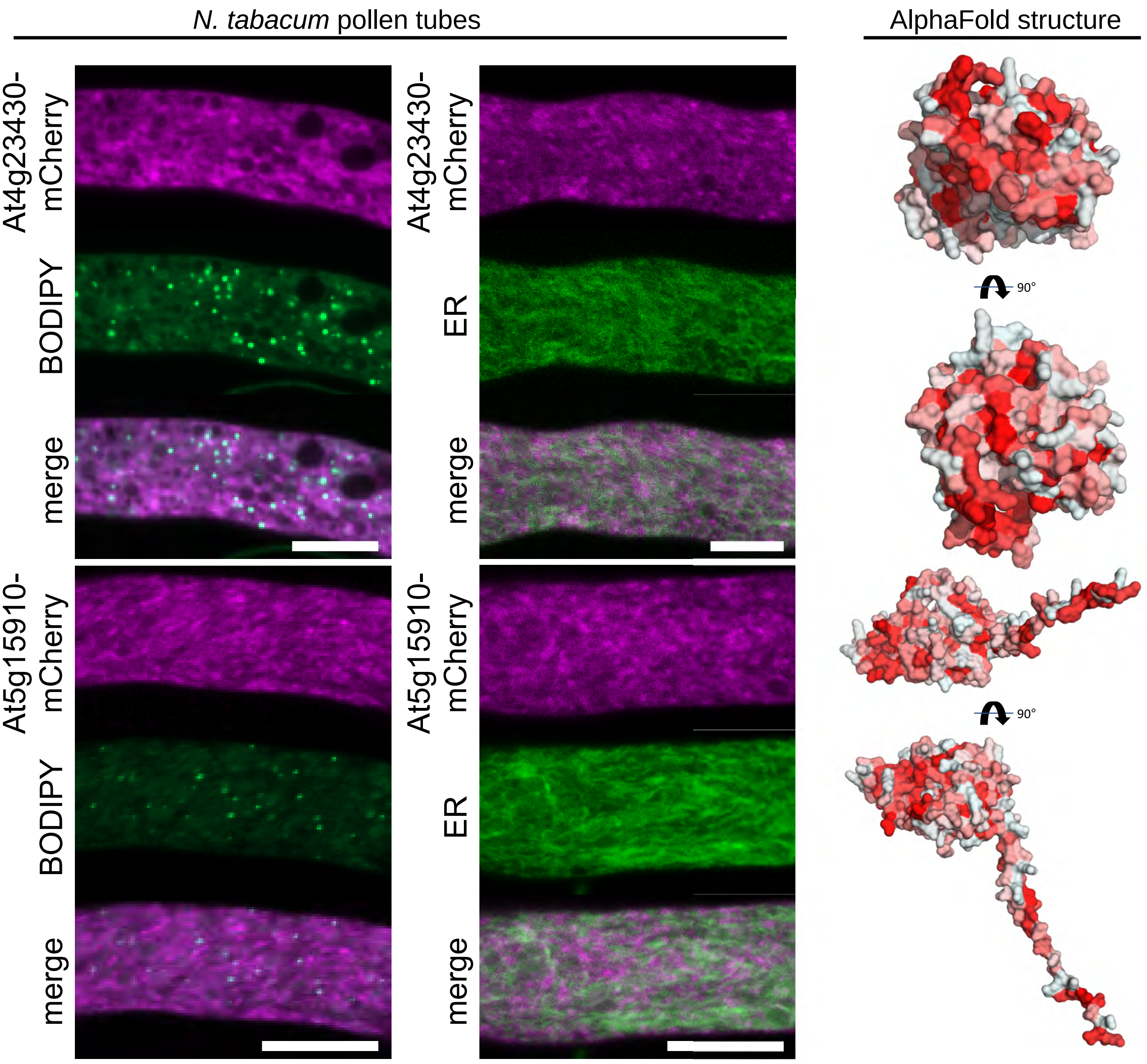

Figure S12: Subcellular localization of Arabidopsis putative dehydrogenases in *N. tabacum* pollen tubes. Indicated mCherry-tagged proteins were expressed in *N. tabacum* pollen tubes. Either LDs were stained with BODIPY 493/503 or ERD2-CFP was co-expressed, serving as an ER marker protein. Images are single planes obtained by CLSM. Both putative dehydrogenases did not colocalize with LDs or the ER. Each image is representative for 6 pollen tubes. Bars, 10 µm. As shown on the right, the structures of both proteins, as predicted by AlphaFold2, show hydrophobic surface regions, but not a flat face as found in several LD-binding proteins.

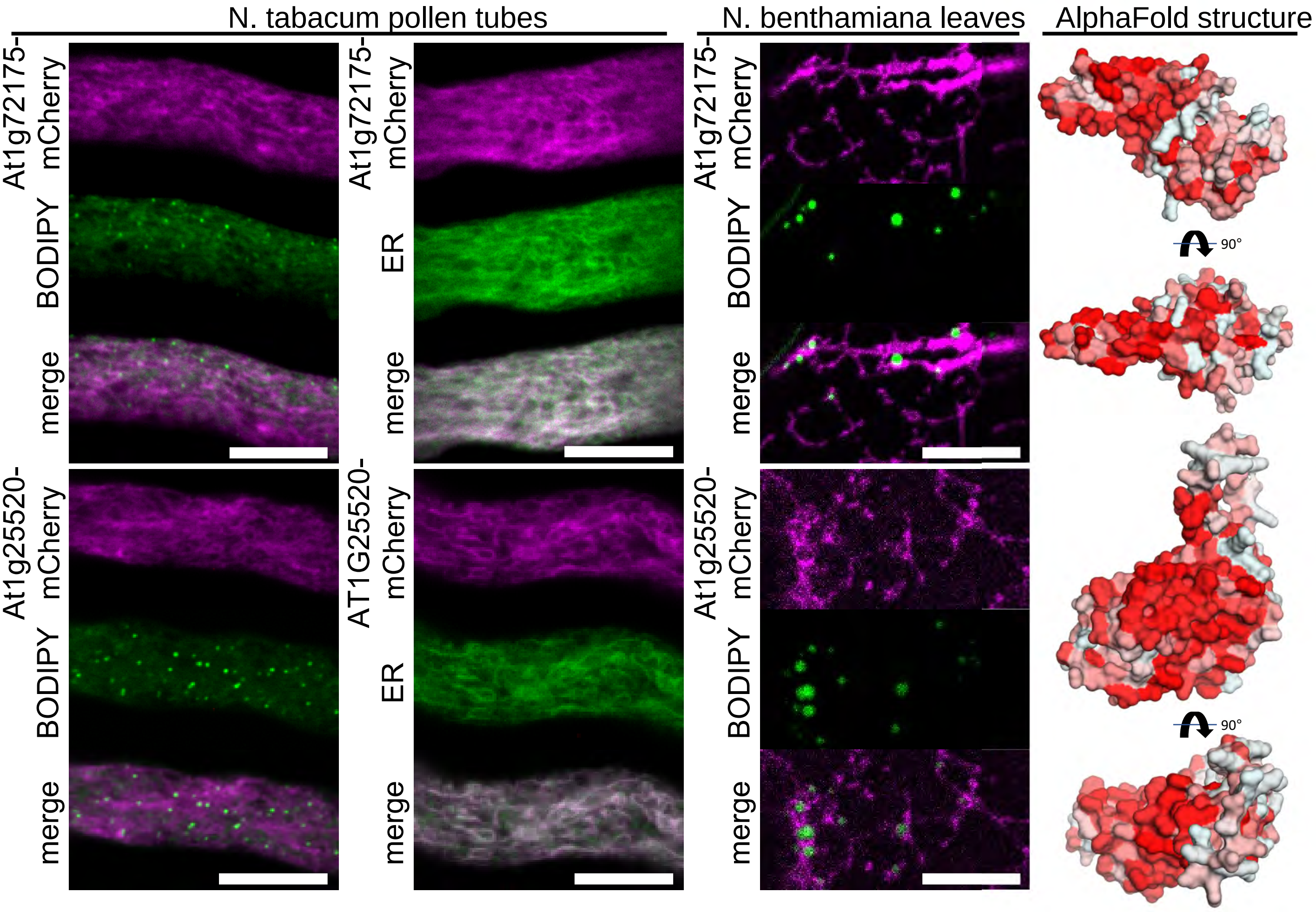

Figure S13: Subcellular localization of selected candidate Arabidopsis root LDs proteins with unknown function in *N. tabacum* pollen tubes and *N. benthamiana* leaf cells. Indicated mCherry-tagged proteins were expressed in either *N. tabacum* pollen tubes or *N. benthamiana* leaves. LDs were stained with BODIPY 493/503 or ERD2-CFP was co-expressed, serving as an ER marker protein. Images are single planes obtained by CLSM. Both the putative zinc finger protein (At1g72175) and a protein of unknown function (PHOTOSYNTHESIS-AFFECTED MUTANT 71 LIKE 4, At1g25520) targeted the ER in pollen tubes and reticular structures in leaves. Each image is representative for at least 9 pollen tubes or 4 leaf areas. Bars, 10 µm. As shown on the right, the structures of both proteins, as predicted by AlphaFold2, show hydrophobic surface regions, but not a flat face as found in several LD-binding proteins.

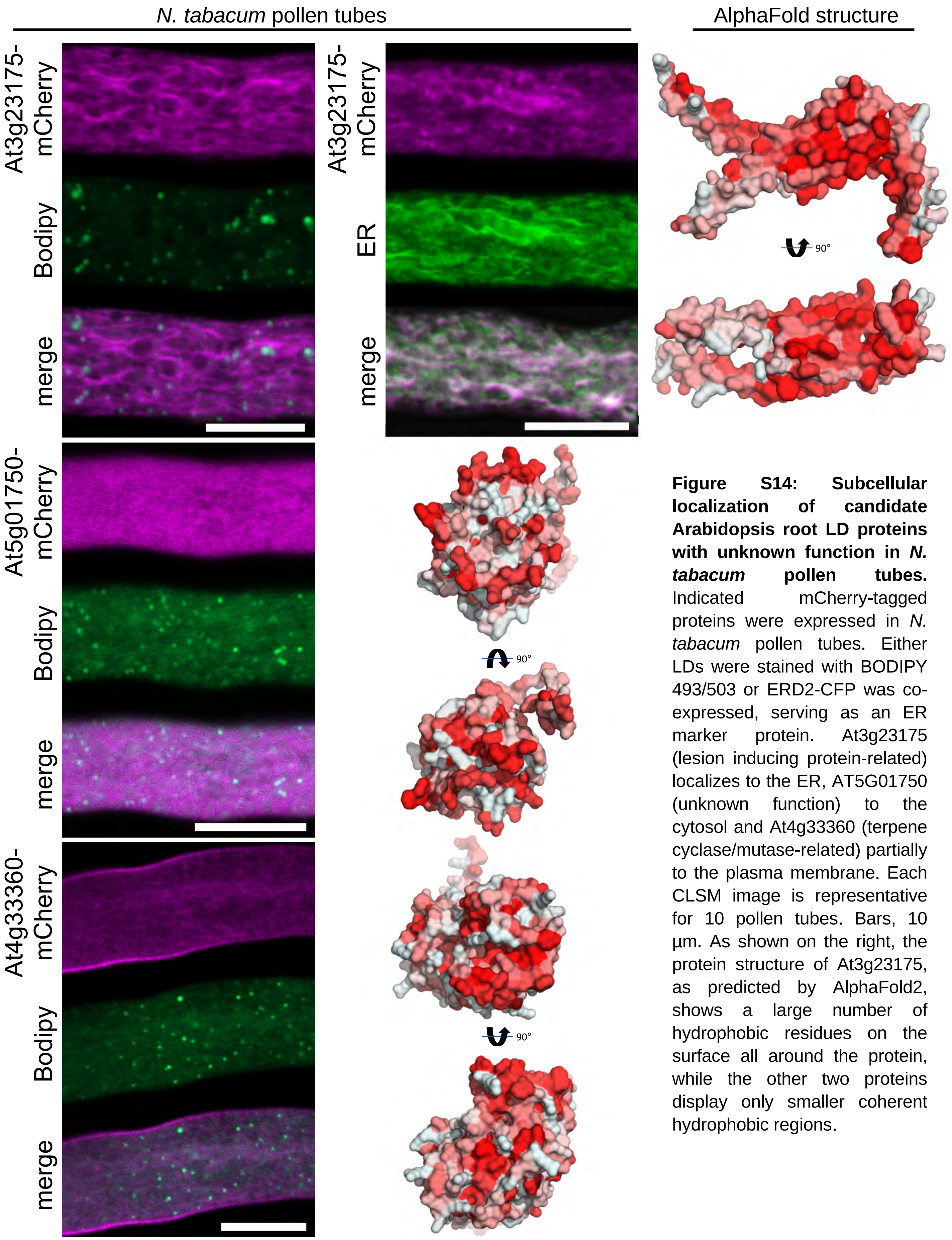

Figure S14: Subcellular localization of candidate Arabidopsis root LD proteins with unknown function in *N. tabacum* pollen tubes. Indicated mCherry-tagged proteins were expressed in *N. tabacum* pollen tubes. Either LDs were stained with BODIPY 493/503 or ERD2-CFP was co-expressed, serving as an ER marker protein. At3g23175 (lesion inducing protein-related) localizes to the ER, AT5G01750 (unknown function) to the cytosol and At4g33360 (terpene cyclase/mutase-related) partially to the plasma membrane. Each CLSM image is representative for 10 pollen tubes. Bars, 10 µm. As shown on the right, the protein structure of At3g23175, as predicted by AlphaFold2, shows a large number of hydrophobic residues on the surface all around the protein, while the other two proteins display only smaller coherent hydrophobic regions.

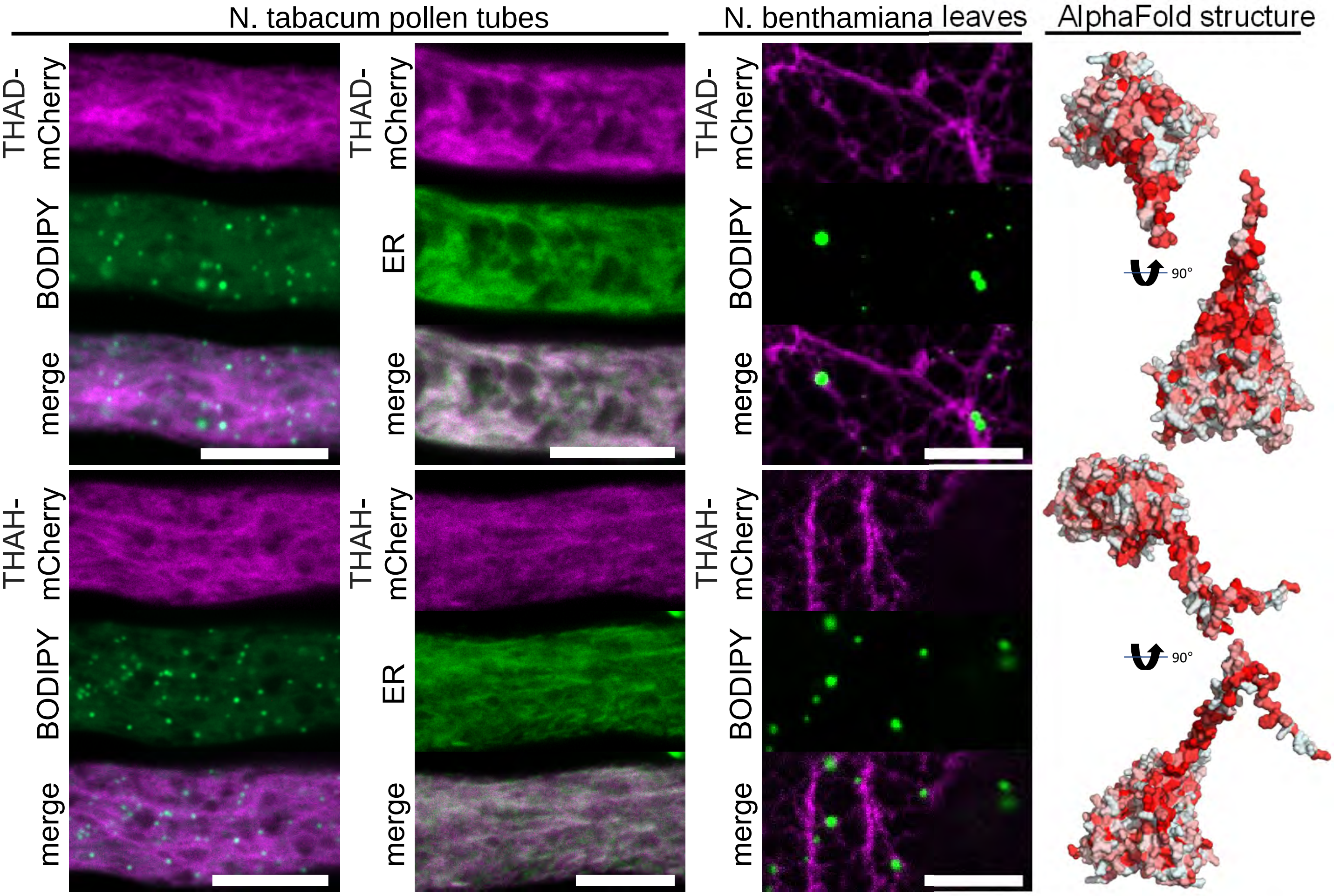

Figure S15: Subcellular localization of various Arabidopsis enzymes acting downstream of thalianol synthase. Indicated mCherry-tagged proteins were expressed in *N. tabacum* pollen tubes or *N. benthamiana* leaves. Either LDs were stained with BODIPY 493/503 or ERD2-CFP was co-expressed, serving as an ER marker protein. Images are single planes obtained by confocal microscopy. Both THALIAN-DIOL DESATURASE (THAD) and THALIANOL HYDROXYLASE (THAD) targeted the ER in pollen tubes and reticular structures in leaves. Each CSLM image is representative for at least 10 pollen tubes or 4 leaf areas. Bars, 10 µm. As shown on the right, the structures of both proteins , as predicted by AlphaFold2, show helical or rod-like hydrophobic regions but no flat hydrophobic surface areas.

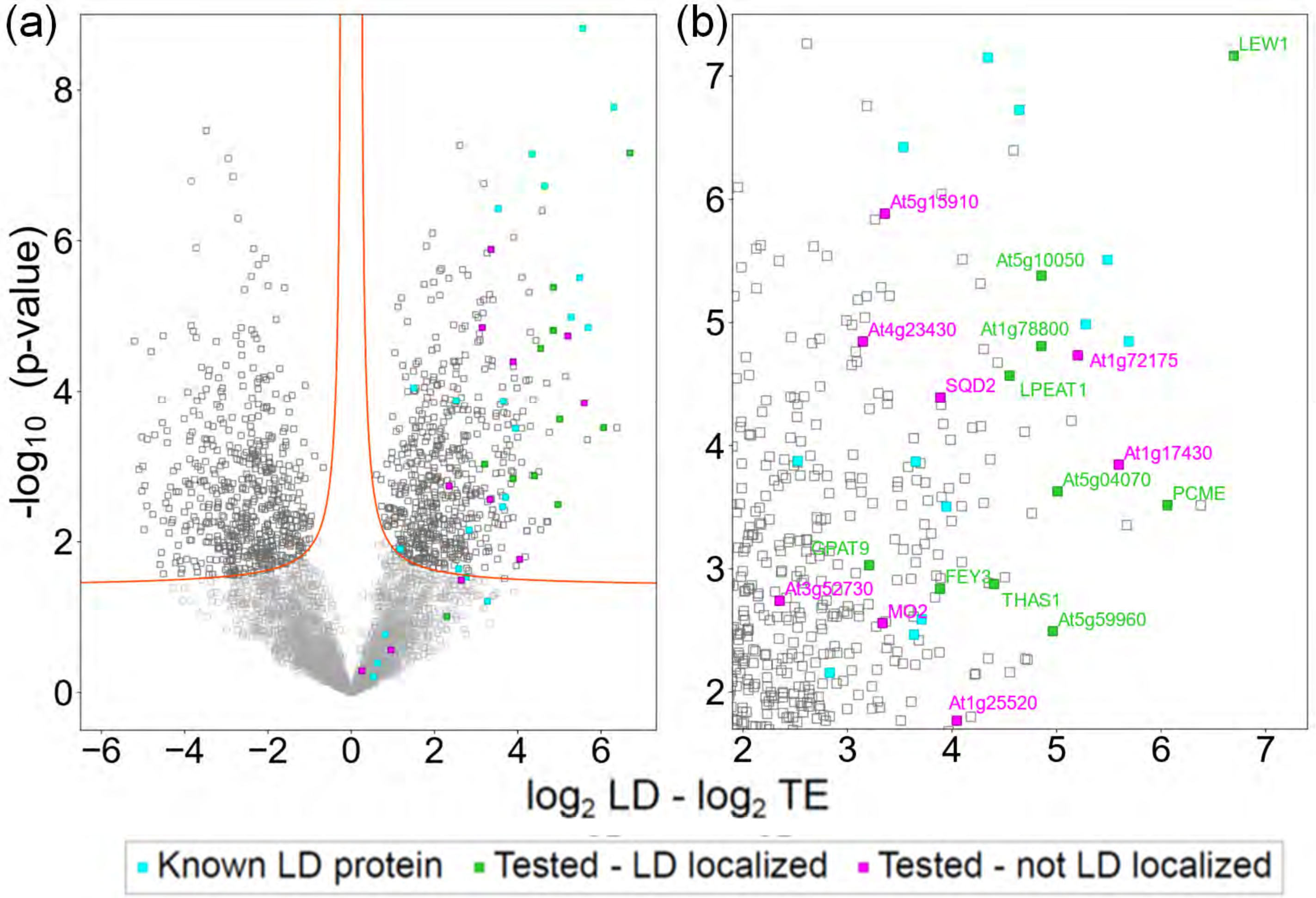

Figure S16: Analysis of protein enrichment in the LD fraction of Arabidopsis roots of the mutant *tgd1-1 sdp1-4* grown in axenic root culture. The riBAQ dataset was imputed and the values were $\log_2$ transformed. Then, the difference between the LD-enriched and total protein fractions was calculated for each protein (Dataset S16). Additionally, the corresponding *p* values ($-\log_{10}$) were determined. (a) A volcano plot was generated based on these values and the upper right corner was enlarged in (b). A false discovery rate (FDR) of 0.01 was used to distinguish between significant and non-significant differences (red lines). Known LD proteins (Table 1) and selected candidate LD proteins (Table 2) are highlighted. FEY3, FOREVER YOUNG 3; GPAT9, GLYCEROL-3-PHOSPHATE ACYLTRANSFERASE9; LPEAT1, LYSOPHOSPHATIDYLETHANOLAMINE ACYLTRANSFERASE1; MO2, MONOOXYGENASE 2; PCME, PRENYLCYSTEINE METHYLESTERASE; SQD2, SULFOQUINOVOSYLDIACYLGLYCEROL 2; THAS1, THALIANOL SYNTHASE 1; TE, total extract.

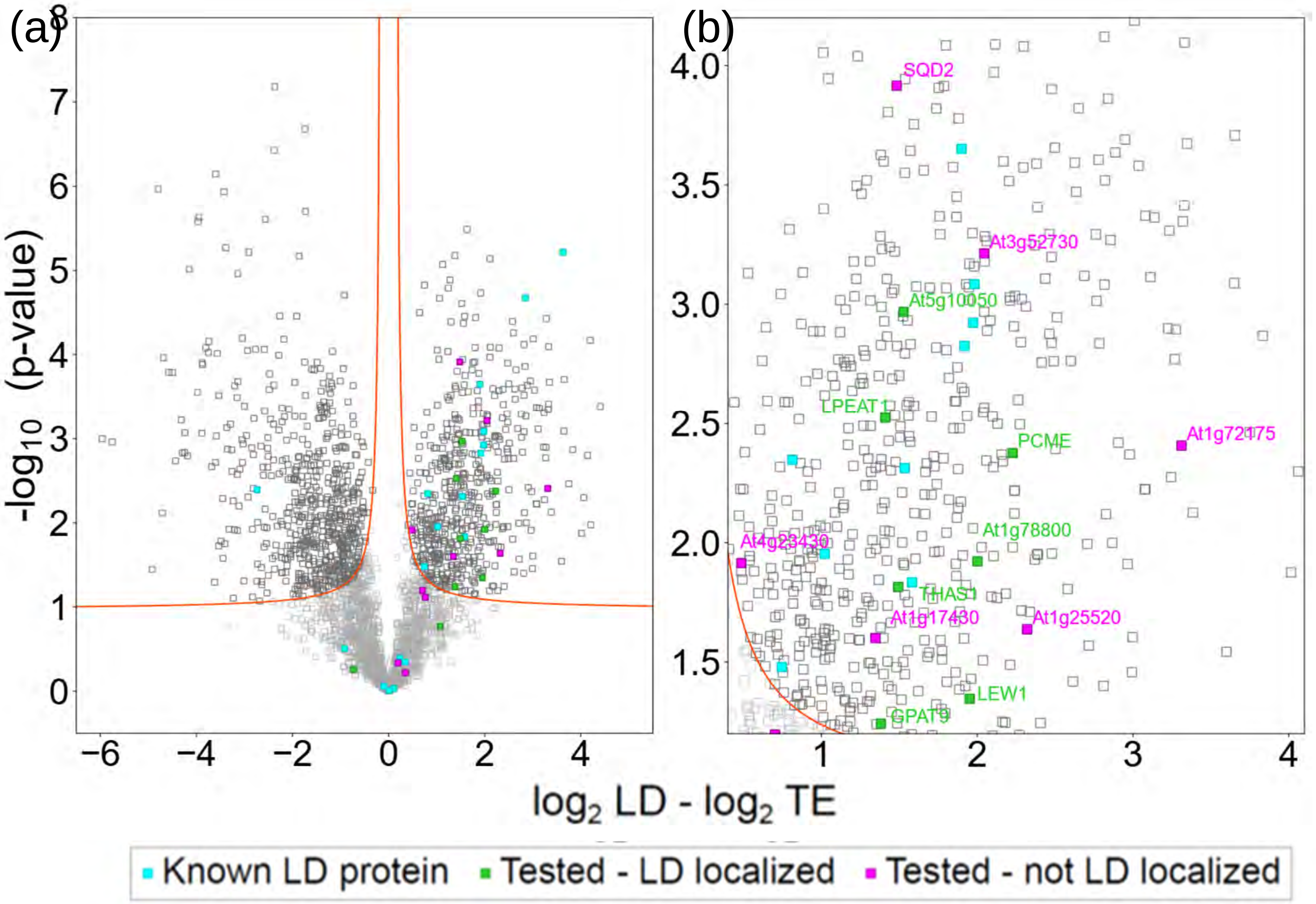

Figure S17: Analysis of protein enrichment in the LD fraction of heat-stressed Arabidopsis roots of the mutant *tgd1-1 sdp1-4* grown in axenic root culture. Flasks containing the root culture were moved to 37°c 24 h prior to harvest. The riBAQ dataset was imputed and the values were log_2 transformed. Then, the difference between the LD-enriched and total protein fractions was calculated for each protein (Dataset S16). Additionally, the corresponding *p* values ($-log_{10}$) were determined. (a) A volcano plot was generated based on these values and the upper right corner was enlarged in (b). A false discovery rate (FDR) of 0.01 was used to distinguish between significant and non-significant differences (red lines). Known LD proteins (Table 1) and selected candidate LD proteins (Table 2) are highlighted. FEY3, FOREVER YOUNG 3; GPAT9, GLYCEROL-3-PHOSPHATE ACYLTRANSFERASE9; LEW1, LEAF WILTING 1; LPEAT1, LYSOPHOSPHATIDYLETHANOLAMINE ACYLTRANSFERASE1; MO2, MONOOXYGENASE 2; PCME, PRENYLCYSTEINE METHYLESTERASE; SQD2, SULFOQUINOVOSYLDIACYLGLYCEROL 2; THAS1, THALIANOL SYNTHASE 1; TE, total extract.

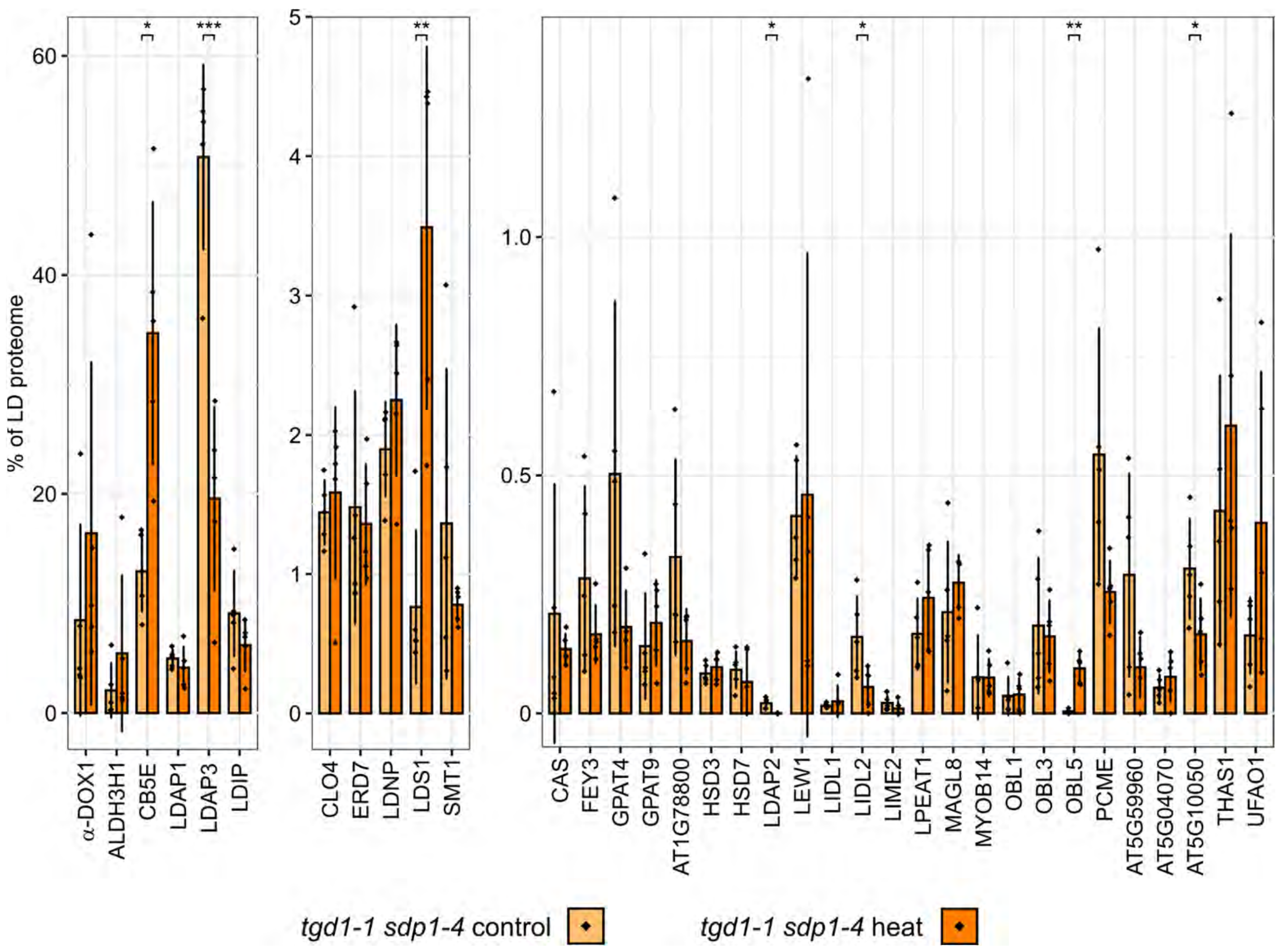

Figure S18: Analysis of protein enrichment in the LD fraction of heat-stressed Arabidopsis roots of the mutant *tgd1-1 sdp1-4* grown in axenic root culture. For heat stress treatment, flasks containing the root culture were moved to 37°c 24 h prior to harvest. The percentage distribution of known LD proteins was calculated for the LD-enriched fractions and compared between control and heat treatment. Values are from n = 5 biological replicates, and are shown as mean ± standard deviation. Statistical differences were calculated by Welch's *t*-test and are represented as follows: $p > 0.05$ "ns", $p < 0.05$ "*", $p < 0.01$ "**", $p < 0.001$ "***".

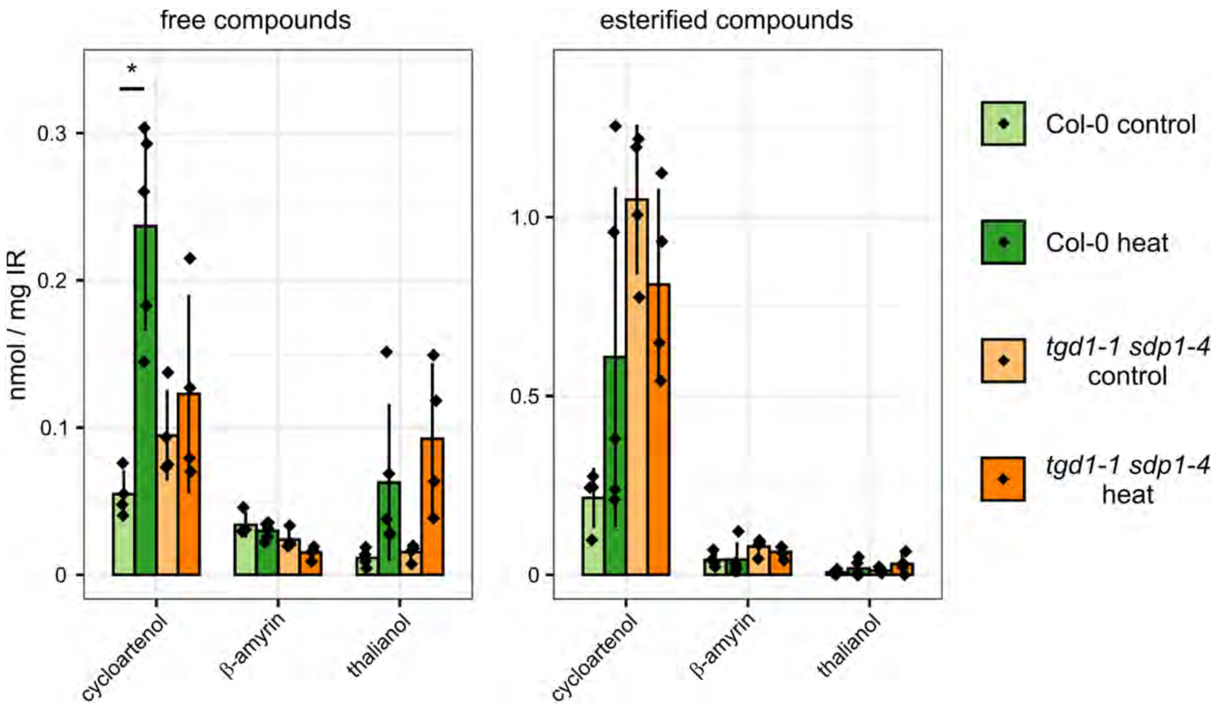

Figure S19: Triterpenoids are found in roots of Arabidopsis Col-0 and the mutant *tdg1-1 sdp1-4*. Cycloartenol, β-amyrin and thalianol and their esterified forms were detected in root lipidomics samples obtained from 12-d-old (Col-0) or 14-d-old (*tdg1-1 sdp1-4*) seedlings after control and heat-stress treatment (same biological samples as presented for lipids in Figure S5 and S6). Values are from n = 4-5 biological replicates, and are shown as mean ± standard deviation. Statistical differences were calculated by Welch's *t*-test using Benjamini-Hochberg correction for multiple comparisons and are represented as follows: $p > 0.05$ "ns", $p < 0.05$ "*". IR, insoluble residue.
