# Supplementary material for: Arabidopsis root lipid droplets are hubs for membrane homeostasis under heat stress, and triterpenoid synthesis and storage
Source: New Phytol. 2025 Nov 25;249(2):892–916. doi: 10.1111/nph.70726 (PMC12712439; doi:10.1111/nph.70726)
Supplement: Supplementary file 6 — Table S1 Metadata for proteomic analysis – unstressed roots. Table S2 Metadata for proteomic analysis – heat‐stressed and unstressed roots. Table S3 List of oligonucleotides used in this study. Table S4 List of microscopy settings for pollen tubes and root LDs. Please note: Wiley is not responsible for the content or functionality of any Supporting Information supplied by the authors. Any queries (other than missing material) should be directed to the New Phytologist Central Office. [file NPH-249-892-s006.pdf]

New Phytologist Supporting Information

Article title: Arabidopsis root lipid droplets are hubs for membrane homeostasis under heat stress, and triterpenoid synthesis and storage.

Authors: Patricia Scholz, Janis Dabisch, Ana C. Vilchez, Alyssa C. Clews, Philipp W. Niemeyer, Magdiel S. S. Lim, Siqi Sun, Lea Hembach, Mayuko Naganawa, Fabienne Dreier, Katharina F. Blersch, Lea M. Preuß, Martin Bonin, Elena Lesch, Yuya Iwai, Takashi L. Shimada, Jürgen Eirich, Iris Finkemeier, Katharina Gutbrod, Peter Dörmann, You Wang, Robert T. Mullen, Till Ischebeck

Article acceptance date: 3 October 2025

Supplemental Tables S1-S4

**Supplemental Table S1: Metadata for proteomic analysis - unstressed roots.** Proteome of *Arabidopsis thaliana tgd1-1 sdp1-4* roots, total cellular extracts and lipid droplet-enriched fractions	
**1. General features**	
Responsible persons	Prof. Till Ischebeck[1], Dr. Jürgen Eirich[2], Prof. Dr. Iris Finkemeier[2]<br>[1]Institute of Plant Biology and Biotechnology (IBBP), University of Münster, Green Biotechnology, Münster 48143, Germany<br>[2]Institute of Plant Biology and Biotechnology (IBBP), University of Münster, Plant Physiology,, Münster 48143, Germany
Instrument manufacturer, model	Thermo Fisher Scientific, Orbitrap Exploris 480
Experimental Design	Analysis of total cellular and lipid droplet-enriched fractions of Arabidopsis *tgd1-1 sdp1-4* roots grown as axenic root culture approach in high-sucrose media
Groups	Total protein, lipid droplet-enriched fraction
Biological and technical replicates	Biological replicates: 5 for each subcellular fraction<br>PS163, 165, … total cellular fractions<br>PS162, 164, … lipid droplet enriched fractions
Sample amount	10
**2. Electrospray Ionisation (ESI)**	
Supply type (static or fed)	fed
Interface manufacturer	Thermo Fisher Scientific
Sprayer type	Nanospray Flex Ion Source
**3.1 Post source component – Analyser**	
	Orbitrap Exploris 480: Orbitrap analyser
**3.2 Post source component – Activation/dissociation**	
Instrument component where the activation/dissociation occurs	Orbitrap Exploris 480: HCD cell
Gas type	Orbitrap Exploris 480: Nitrogen
Activation/dissociation type	Orbitrap Exploris 480: HCD
**4.1 Spectrum and peak list generation and annotation – Data acquisition**	
Software name and version	Xcalibur 4.0
Acquisition parameters	Data-dependent Top20
Software name and version	MaxQuant 1.6.2.17
**4.2 Spectrum and peak list generation and annotation – Resulting data**	
Location of source and processed files	Proteomic raw data can be found in the PRIDE database under the identifier PXD051152 (https://www.ebi.ac.uk/pride/).
**5. Description of the software and methods applied in the quantitative analysis**	
Quantification software	MaxQuant 1.6.2.17
Description of the selection and/or	Upload of all .raw files into the software. Grouping of technical replicates as one Experiment (“set experiment”).

matching method of features, together with the description of the method of the primary extracted quantification values determination for each feature and/or peptide	Group-specific parameters:<br>1) Type: default<br>2) Digestion: default<br>3) Modifications: default<br>4) Label-free quantification: LFQ, default<br>5) Instrument: intensity determination: total sum, rest default<br>6) First search: default<br>7) Misc: default<br>Global parameters<br>1) Sequences: updated TAIR10 peptides from 14.12.2010, rest default<br>2) Identification: PSM FDR=0.01, protein FDR=0.01, Match between runs ✓, rest default<br>3) Protein quantification: default<br>4) Label free quantification: iBAQ ✓, rest default<br>5) Tables: default<br>6) Folder locations: default<br>7) MS/MS analyzer: FTMS recalibration ✓, rest default<br>8) Advanced: default
Confidence filter of features or peptides prior to quantification	Global parameters: identification: PSM FDR=0.01, protein FDR=0.01, rest default
Normalisation	All values were divided by the total iBAQ intensities or total LFQ intensities in one sample and multiplied by 1000.

**Supplemental Table S2: Metadata for proteomic analysis – heat-stressed and unstressed roots.** Proteome of *Arabidopsis thaliana tgd1-1 sdp1-4* roots, total cellular extracts and lipid droplet-enriched fractions. Roots were either treated for 24 h at 37° C or remained at abient temperature (control)	
**1. General features**	
Responsible persons	Prof. Till Ischebeck[1], Dr. Jürgen Eirich[2], Prof. Dr. Iris Finkemeier[2]<br>[1]Institute of Plant Biology and Biotechnology (IBBP), University of Münster, Green Biotechnology, Münster 48143, Germany<br>[2]Institute of Plant Biology and Biotechnology (IBBP), University of Münster, Plant Physiology,, Münster 48143, Germany
Instrument manufacturer, model	Thermo Fisher Scientific, Orbitrap Exploris 480
Experimental Design	Analysis of total cellular and lipid droplet-enriched fractions of Arabidopsis *tgd1-1 sdp1-4* roots grown as axenic root culture approach in high-sucrose media
Groups	Total protein, lipid droplet-enriched fraction
Biological and technical replicates	Biological replicates: 5 for each subcellular fraction and treatment<br>KT229, 231, … total cellular fractions control<br>KT239, 241, … lipid droplet enriched fractions control<br>KT230, 232, … total cellular fractions heat<br>KT240, 242, … lipid droplet enriched fractions heat
Sample amount	20
**2. Electrospray Ionisation (ESI)**	
Supply type (static or fed)	fed
Interface manufacturer	Thermo Fisher Scientific
Sprayer type	Nanospray Flex Ion Source
**3.1 Post source component – Analyser**	
	Orbitrap Exploris 480: Orbitrap analyser
**3.2 Post source component – Activation/dissociation**	
Instrument component where the activation/dissociation occurs	Orbitrap Exploris 480: HCD cell
Gas type	Orbitrap Exploris 480: Nitrogen
Activation/dissociation type	Orbitrap Exploris 480: HCD
**4.1 Spectrum and peak list generation and annotation – Data acquisition**	
Software name and version	Xcalibur 4.0
Acquisition parameters	Data-dependent Top20
Software name and version	MaxQuant 1.6.2.17
**4.2 Spectrum and peak list generation and annotation – Resulting data**	
Location of source and processed files	Proteomic raw data can be found in the PRIDE database under the identifier PXD068568 (https://www.ebi.ac.uk/pride/).
**5. Description of the software and methods applied in the quantitative analysis**	

Quantification software	MaxQuant 1.6.2.17
Description of the selection and/or matching method of features, together with the description of the method of the primary extracted quantification values determination for each feature and/or peptide	Upload of all .raw files into the software. Grouping of technical replicates as one Experiment ("set experiment").<br>Group-specific parameters:<br>1) Type: default<br>2) Digestion: default<br>3) Modifications: default<br>4) Label-free quantification: LFQ, default<br>5) Instrument: intensity determination: total sum, rest default<br>6) First search: default<br>7) Misc: default<br>Global parameters<br>1) Sequences: updated TAIR10 peptides from 14.12.2010, rest default<br>2) Identification: PSM FDR=0.01, protein FDR=0.01, Match between runs ✓, rest default<br>3) Protein quantification: default<br>4) Label free quantification: iBAQ ✓, rest default<br>5) Tables: default<br>6) Folder locations: default<br>7) MS/MS analyzer: FTMS recalibration ✓, rest default<br>8) Advanced: default
Confidence filter of features or peptides prior to quantification	Global parameters: identification: PSM FDR=0.01, protein FDR=0.01, rest default
Normalisation	All values were divided by the total iBAQ intensities or total LFQ intensities in one sample and multiplied by 1000.

Supplemental table S3. List of oligonucleotides used in this study.

AGI	Acronym	Name	5' end	3' end
AT5G60620	GPAT9	glycerol-3-phosphate acyltransferase 9	GGGG ACAAGTTTGTACAAAAAAGCAGGCT C ATGAGCAGTACGGCAGGG	GGGG ACCACTTTGTACAAGAAAGCTGGGT CCTTCTCTTCCAATCTAGCCAGGA
AT3G11430	GPAT5	GLYCEROL-3-PHOSPHATE sn-2-ACYLTRANSFERASE 5	GGGG ACAAGTTTGTACAAAAAAGCAGGCT C ATGGTTATGGAGCAAGCTGGAAC	GGGG ACCACTTTGTACAAGAAAGCTGGGT C ATGGAGACAAGGCTCGAAAGTG
AT1G01610	GPAT4	GLYCEROL-3-PHOSPHATE sn-2-ACYLTRANSFERASE 4	GGGG ACAAGTTTGTACAAAAAAGCAGGCT C ATGTCTCCGGCGAAGAAGA	GGGG ACCACTTTGTACAAGAAAGCTGGGT C CTCCATGGACTTGGTCTTATTGAT
AT1G80950	LPEAT1	LYSOPHOSPHATIDYLETHANOLAMINE ACYLTRANSFERASE1	GGGG ACAAGTTTGTACAAAAAAGCAGGCT C ATGGAATCAGAGCTCAAAGATTTGAA	GGGG ACCACTTTGTACAAGAAAGCTGGGT C TTCTTCTTTCTGATGGAAATCACGG
AT5G10050		putative short-chain dehydrogenase	GGGG ACAAGTTTGTACAAAAAAGCAGGCT C ATGGAGAGTGGCGATGAGAGTC	GGGG ACCACTTTGTACAAGAAAGCTGGGT C CTTCTTCATTAAAACCTGCTTCTGG
AT5G04070		putative short-chain dehydrogenase	GGGG ACAAGTTTGTACAAAAAAGCAGGCT C ATGGAGAATTTGAAGGAGGCT	GGGG ACCACTTTGTACAAGAAAGCTGGGT C AGTGTGAGTTTGCTGCAATT
AT1G44170		putative aldehyde dehydrogenase	GGGG ACAAGTTTGTACAAAAAAGCAGGCT C ATGGCTGCGAAGAAGGTTTTTG	GGGG ACCACTTTGTACAAGAAAGCTGGGT C AGCTAAACCGAGAAGGACTTTG
AT1G78800		glycosyl transferase family (gateway)	GGGG ACAAGTTTGTACAAAAAAGCAGGCT C ATGGCGAAAAAAGAAGGTTCAAAG	GGGG ACCACTTTGTACAAGAAAGCTGGGT C ATCTTCTTTAGGACTTGATACGAC
AT1G16570		putative N-glycan biosynthetic enzyme	GCAGGCTCCGCGGCCATGGGGAAAAGAGGAAGGGCT	AGCTGGGTCGGCGCGTGAATCTGCAATTTGAGACAC
AT1G78800		glycosyl transferase family	GCAGGCTCCGCGGCCATGGCGAAAAAAGAAGGTTCA	AGCTGGGTCGGCGCGATCTTCTTTAGGACTTGATAC
AT2G47760		putative N-glycan biosynthetic enzyme	GCAGGCTCCGCGGCCATGGCGGGCGCCTCATCACCG	AGCTGGGTCGGCGCGTGCTTTTTTGTGTATTTGGGA
AT5G38460		putative N-glycan biosynthetic enzyme	GCAGGCTCCGCGGCCATGCCGAAGAAGAAGCCGGCG	AGCTGGGTCGGCGCGGATTTGCTTCTTTTCTTTATC
AT2G40190		putative N-glycan biosynthetic enzyme	GCAGGCTCCGCGGCCATGGCGATCTACTTCATTCTC	AGCTGGGTCGGCGCGTTTAAGAGGACCTGTGAAAAT
AT5G15860	PCME	prenylcysteine methylesterase	GGGG ACAAGTTTGTACAAAAAAGCAGGCT C ATGCATTCGCCTCTTCAGACTC	GGGG ACCACTTTGTACAAGAAAGCTGGGT C GAAAGGGCTAATCTCACGAGCC
AT4G27760	FEY3	FOREVER YOUNG	GGGG ACAAGTTTGTACAAAAAAGCAGGCT C ATGAGTGACGAAACGACGTCATC	GGGG ACCACTTTGTACAAGAAAGCTGGGT C TTCGTGTTGTGCTCCATACCG
AT4G33180		putative hydrolase	GGGG ACAAGTTTGTACAAAAAAGCAGGCT C ATGTCGCTGTCGTCTTACCTC	GGGG ACCACTTTGTACAAGAAAGCTGGGT C AATATTGTTGAACTCTTGAGCACATTC
AT4G13160	MYOB14	MYOSIN BINDING PROTEIN 14	GGGG ACAAGTTTGTACAAAAAAGCAGGCT C ATGGACTACCAAGAAAGTTATAGATTGAC	GGGG ACCACTTTGTACAAGAAAGCTGGGT C TGGGAGATGTGTTGAAGATGAAGT
AT1G30130	UFAO1	UNSATURATED FATTY ACID OXIDASE 1	GGGG ACAAGTTTGTACAAAAAAGCAGGCT C ATGGAATTGCTCTTTCTCCTCTGT	GGGG ACCACTTTGTACAAGAAAGCTGGGT C TGACCAAGGCCAGTTCGCAT
AT5G59960		protein of unkknown function	GGGG ACAAGTTTGTACAAAAAAGCAGGCT C ATGGAGAAAGTTTCGTCGCCG	GGGG ACCACTTTGTACAAGAAAGCTGGGT C GCTCTGCCTTGGCTTTTCTCG
AT1G11755	LEW1	LEAF WILTING 1	GGGG ACAAGTTTGTACAAAAAAGCAGGCT C ATGGATTCGAATCAATCGATG	GGGG ACCACTTTGTACAAGAAAGCTGGGT C AGTTCCATAGTTTTGGTGGAC
AT5G48010	THAS1	THALIANOL SYNTHASE 1	GGGG ACAAGTTTGTACAAAAAAGCAGGCT C ATGTGGAGGCTGAGAACTG	GGGG ACCACTTTGTACAAGAAAGCTGGGT C AGGGAGGAGACGTCGC
AT5G42600	MRN1	marneral synthase 1	GGGG ACAAGTTTGTACAAAAAAGCAGGCT C ATGTGGAGACTGCGAATTGGAGC	GGGG ACCACTTTGTACAAGAAAGCTGGGT C AGAAACAAGCAGACGCAGAGC
AT4G38540	MO2	monooxygenase 2	GGGG ACAAGTTTGTACAAAAAAGCAGGCT C ATGGAAGAAGAAGGCAGCCC	GGGG ACCACTTTGTACAAGAAAGCTGGGT C TGGGACAAGGCTTCCGC
AT1G17430		putative hydrolase	GGGG ACAAGTTTGTACAAAAAAGCAGGCT C ATGGCGTCCATGAAACACG	GGGG ACAAGTTTGTACAAAAAAGCAGGCT C AGCACTTAAGACAAACGAAG
AT5G01220	SQD2	sulfoquinovosyldiacylglycerol 2	GGGG ACAAGTTTGTACAAAAAAGCAGGCT C ATGACGACTCTTTCTTCTATAAATC	GGGG ACCACTTTGTACAAGAAAGCTGGGT C CACGTTACCTTCCGGTACTGG
AT4G23430		putative short-chain dehydrogenase	GGGG ACAAGTTTGTACAAAAAAGCAGGCT C ATGTGGTTTTTTGGATCGAAAG	GGGG ACAAGTTTGTACAAAAAAGCAGGCT C AGAACTGCTTTCTCCTGATTG
AT5G15910		putative short-chain dehydrogenase	GGGG ACAAGTTTGTACAAAAAAGCAGGCT C ATGTTAAGGTCTCTGATTTGG	GGGG ACAAGTTTGTACAAAAAAGCAGGCT C GTGACCATGTTGAAGAATCC
AT1G72175		putative zinc finger protein	GGGG ACAAGTTTGTACAAAAAAGCAGGCT C ATGAATAGTCCACCGGAGAAC	GGGG ACCACTTTGTACAAGAAAGCTGGGT C CGAACCGGCATGACGGAAAT
AT1G25520	PML4	photosynthesis-affected mutant 71 like 4	GGGG ACAAGTTTGTACAAAAAAGCAGGCT C ATGAGCTCGGTTTTGCAGGG	GGGG ACCACTTTGTACAAGAAAGCTGGGT C AGCCTCAACAGAAGTAAGATACG
AT3G23175		lesion inducing protein-related	GGGG ACAAGTTTGTACAAAAAAGCAGGCT C ATGAAGATGTCAATTAGCAAGGTC	GGGG ACCACTTTGTACAAGAAAGCTGGGT C TCCTTCGGTCTGCTTGTTCTTC
AT5G01750		unknown function	CTTTGTACAAAAAAGCAGGCT C ATGGAGCAGCCGTACGTGTAC	CTTTGTACAAGAAAGCTGGGT C GAGATAGTATACTTGGGCAACAATGGC
AT4G33360	FLDH	farnesol dehydrogenase	GGGG ACAAGTTTGTACAAAAAAGCAGGCT C ATGGGCCCAAAGATGCCCAAC	GGGG ACCACTTTGTACAAGAAAGCTGGGT C GTAGTGAATGACGCCCAGACT
AT5G47990	THAD	THALIAN-DIOL DESATURASE	GGGG ACAAGTTTGTACAAAAAAGCAGGCT C ATGGCATCAATGATCACTGTTGAC	GGGG ACCACTTTGTACAAGAAAGCTGGGT C AGTGTTTAGGTTTCGAGGAACAG
AT5G48000	THAH	THALIANOL HYDROXYLASE	GGGG ACAAGTTTGTACAAAAAAGCAGGCT C ATGGATCGACGGGGCAAGTAT	GGGG ACCACTTTGTACAAGAAAGCTGGGT C GAGTGACTGGGAAATCTTGATAG

Table S3. List of microscopy settings for pollen tubes and root LDs. All proteins were labeled with mCherry except for the ER marker ERD2 that was labeled with CFP. All LDs were stained with BODIPY 493/503.

Microscopy of	405 laser	445 laser	458 laser	488 laser	561 laser	594 laser	detector "ERD-CF	detector BODIPY 49	detector mCherry
At1g72175, At3g52730, At4g38540	Y	N	N	Y	N	Y	464-508	499-535	597-650
At1g11755	N	Y	N	Y	Y	N	455-481	508-535	600-640
At1g25520, At1g44170, At4g27760, At5g04070, At5g10050, At5g15860, At5g59960, At1g78800, At4g23430, At4g33180, At5g15910	N	Y	N	N	Y	N	455-481	n.a.	597-641
At5g47990, At5g48000	N	N	Y	N	Y	N	463-552	n.a.	568-620
At3g11430	Y	N	N	Y	N	Y	410-659	499-579	597-695
At1g11755	N	N	N	Y	Y	N	n.a.	490-539	600-640
At1g25520, At1g44170, At4g27760, At5g47990, At5g48000, At1g17430, At4g00550, At4g33180, At4g33360, At5g01220, At3g23175	N	N	N	Y	Y	N	n.a.	493-544	578-696
At3g11430, At5g59960, At4g02370, At4g23430, At5g15910	N	N	N	Y	Y	N	n.a.	499-535	579-659
At3g52730, At1g01610, At1g80950, At4g13160, At5g48010, At5g60620, At4g38540	N	N	N	Y	Y	N	n.a.	497-542	590-640
At5g04070	N	N	N	Y	Y	N	n.a.	490-535	600-640
At5g10050, At5g15860	N	N	N	Y	Y	N	n.a.	490-539	585-658
At1g30130	N	N	N	Y	Y	N	n.a.	490-539	600-690
At1g78800	N	N	N	Y	Y	N	n.a.	499-535	597-641
roots	N	N	N	Y	N	N	n.a.	495-550	n.a.
